# Replantation of a displaced incisor in a boy with a cleft lip and alveolus: a case report

**DOI:** 10.1186/s13256-016-1041-1

**Published:** 2016-09-15

**Authors:** Heidrun Schaaf, Heiko Kerkmann, Felix Pitka, Niko Christian Bock, Sameh Attia

**Affiliations:** 1 Maxillofacial Surgery MKG am Theater, Neuenweg 16, 35390 Giessen, Germany; 2 Department of Orthodontics, University of Giessen, Schlangenzahl 14, 35392 Giessen, Germany; 3 Department of Maxillofacial Surgery, University of Giessen, Schlangenzahl 14, 35392 Giessen, Germany

**Keywords:** Auto-alloplastic replantation, Ectopic incisor, Autotransplantation, Cleft lip

## Abstract

**Background:**

Tooth replantation after traumatic avulsion or transplantation is a challenge in oral surgery. A special method named auto-alloplastic replantation, in which a titanium post is used after extraoral endodontic treatment, combines several advantages. The treatment is performed in one step with no further endodontic intervention, no wide root canal instrumentation, no recontamination, and reduced endodontic infection. This is the first report on replantation of an ectopic tooth in a patient with a cleft lip and alveolus using this method.

**Case presentation:**

This case report presents the treatment of a 13-year-old white boy with a cleft lip and alveolus who had an ectopic incisor in the cleft region. His rehabilitation was performed by a tooth transposition using the auto-alloplastic replantation technique. After preparation of the displaced incisor from the vestibule, extraoral endodontic treatment followed using a titanium post prior to replantation in a newly formed socket. In the follow-up, the tooth is still in place and functioning after 2 years.

**Conclusions:**

This method can be used to bridge the years while a patient is young and jaw growth is incomplete until bone augmentation and implantation can be performed. The tooth will preserve the remaining alveolar ridge and help the adolescent psychologically.

## Background

Ectopic development of teeth is known in patients with a cleft lip and alveolus for various reasons. Teeth can appear in different locations in the mouth, the nose, and the sinus. Rare abnormal positions have been reported in the nose [1, 2] or in the maxillary sinus [3]. These teeth cannot be preserved and must be removed.

Cases with tooth transplantation in patients with a cleft lip and alveolus are rare, but a case has been reported on a secondary bone graft with simultaneous auto-tooth transplantation [4]. The positive effect of a tooth in the alveolar ridge is to stabilize the maxillary arch. Furthermore, dental roots support the growing maxilla and the transplantation of a tooth prevents reabsorption of the bone. In a case series about the indications for the use of autotransplantation of teeth, one case describes a successful transplantation of the left permanent canine into the position of the dilacerated maxillary left permanent central incisor region [5]. Another successful autotransplantation of a premolar to the incisor region in a patient with a cleft lip and alveolus is described 6 months after bone grafting the defect in the cleft area [6]. Furthermore, a systematic review and meta-analysis on tooth autotransplantation found an excellent survival rate that ranged from 75.3 to 91 % [7]. In a study, 215 patients were examined after autotransplantation with a median follow-up of 4.8 years and showed a success rate of 81 %; the highest success rate was in the maxillary incisor region with 100 % [8]. Specific knowledge concerning the frontal region was presented for the autotransplantation of premolars to the maxillary incisor region after follow-up of 12 to 22 years [9].

Tooth replantation can be considered an effective alternative to dental implant, when the latter is contraindicated in a young patient with incomplete skeletal development [10] or in special cases with dentigerous cyst [11]. In dental traumatology, there is a special method for the treatment of avulsed or severely traumatized incisors: auto-alloplastic replantation. Auto-alloplastic replantation of a tooth describes transplantation with vital periodontal ligament and an alloplastic retrograde titanium post after extraoral endodontic treatment and antireabsorptive-regenerative therapy [12, 13]. The indication for this treatment protocol includes the replantation of avulsed teeth or transplantation of primary canines [14]. For later prosthetic rehabilitation of a missing tooth in patients with a cleft lip and alveolus, dental implants are a feasible solution [15, 16]. Implants can replace a single tooth, mostly the lateral incisor in the cleft area, or can support a dental prosthesis if more than one tooth is missing.

Due to the fact that there are only a handful of publications about auto-replantation of a tooth in patients with a cleft lip and alveolus, it is necessary to communicate these alternative treatment methods and their outcome.

## Case presentation

**Fig. 1:**
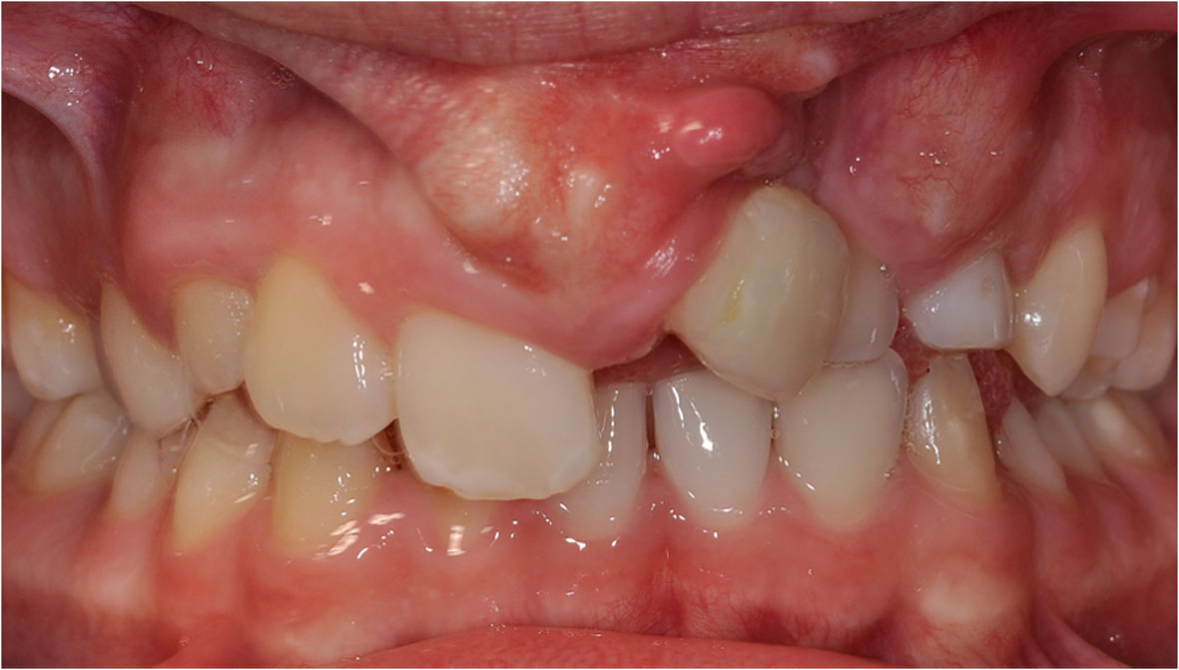
Patient with a unilateral cleft. Left middle incisor in an ectopic position in the frontal vestibule

A 13-year-old white boy born with a complete unilateral cleft lip and alveolus was receiving orthodontic treatment. He was referred for surgical treatment. A clinical examination showed his left middle incisor in an ectopic position in his frontal vestibule. The root was twisted to the midline and the crown was heavily displaced to the lateral side (Fig. 1). The occlusion was not acceptable. An orthodontic tooth movement would be too difficult and cause side effects, such as periodontal problems, or reabsorption of the root or adjacent permanent teeth. An immediate removal of the tooth and insertion of a dental implant was not the treatment of choice due to his young age and incomplete jaw growth. Our main long-term aim was to preserve as much bone as possible in his compromised cleft area.

The psychological aspect of this condition should not be underestimated because he was ashamed of his front teeth and was not able to smile. He describes the appearance of his maxillary anterior teeth as unaesthetic. In cooperation with the Department of orthodontics, a decision for surgical intervention was made.

**Fig. 2:**
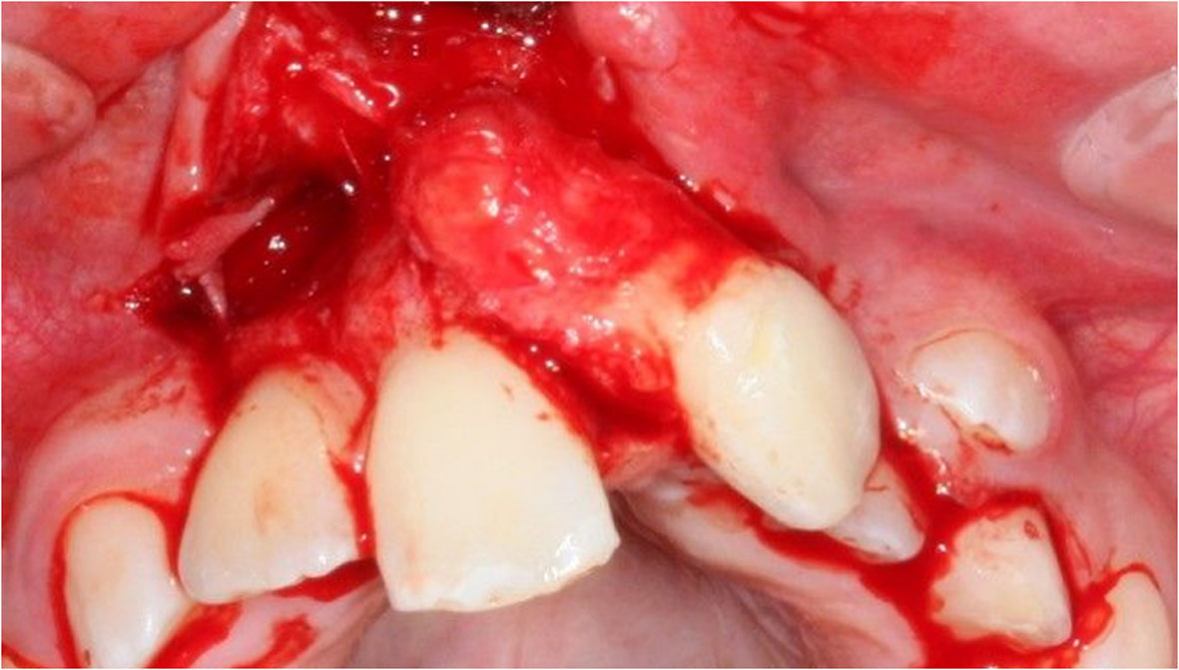
Intraoperative view showing careful preparation of the tooth to preserve the periodontal ligament

**Fig. 3:**
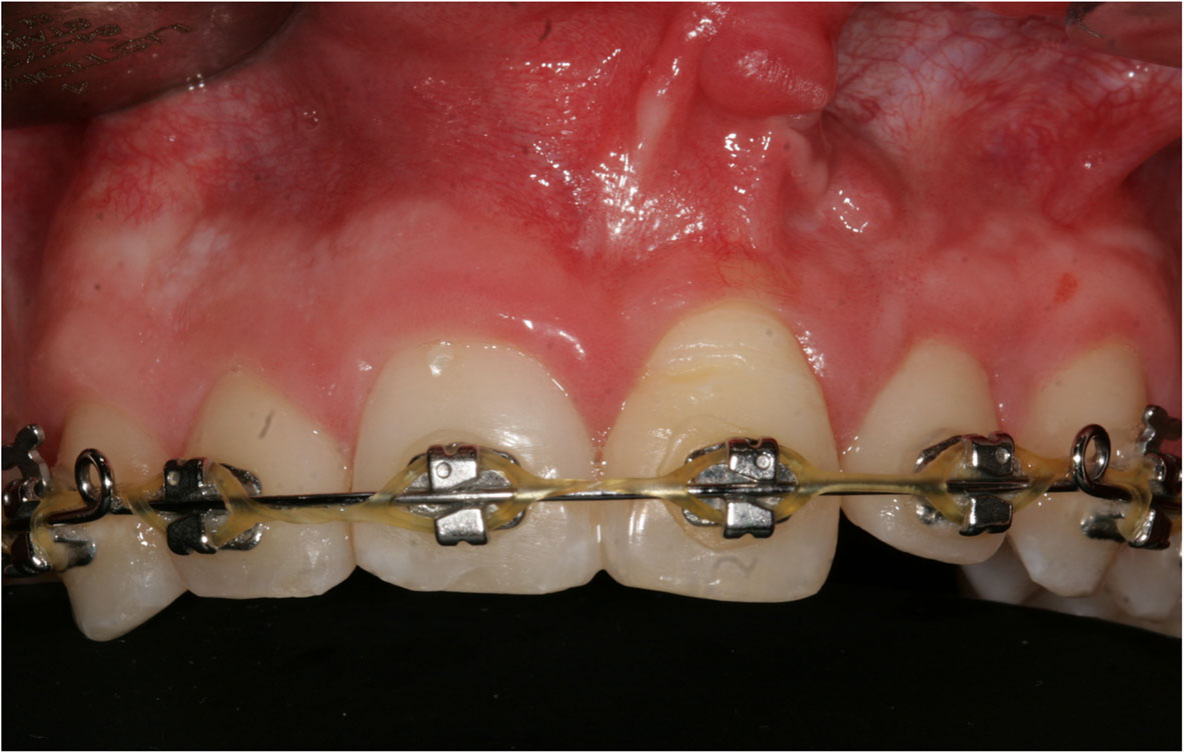
Photograph taken 24 months after autotransplantation of middle incisor from the vestibule into the newly formed socket

**Fig. 4:**
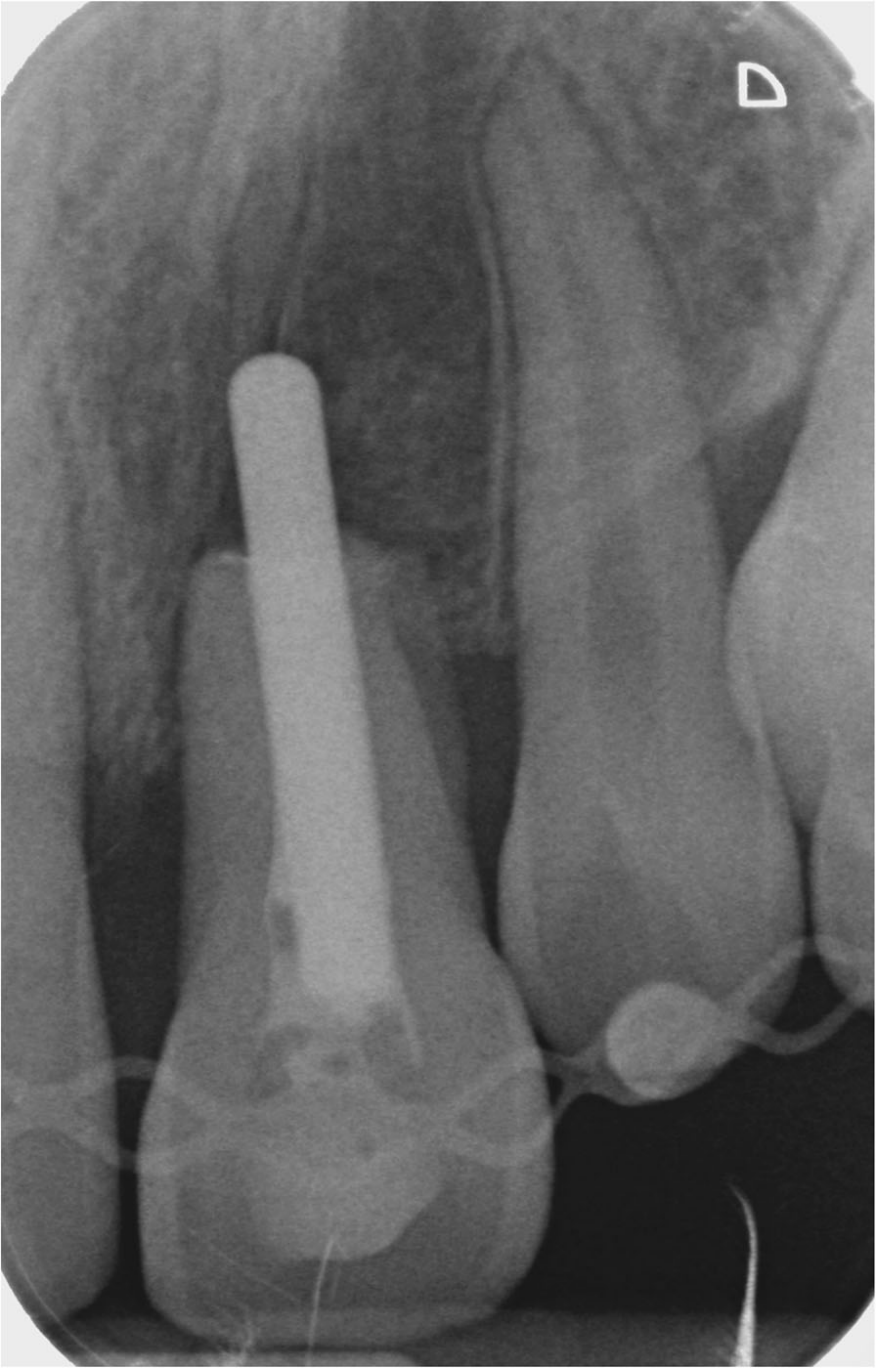
Radiograph showing the middle incisor directly after transplantation and splinting

**Fig. 5:**
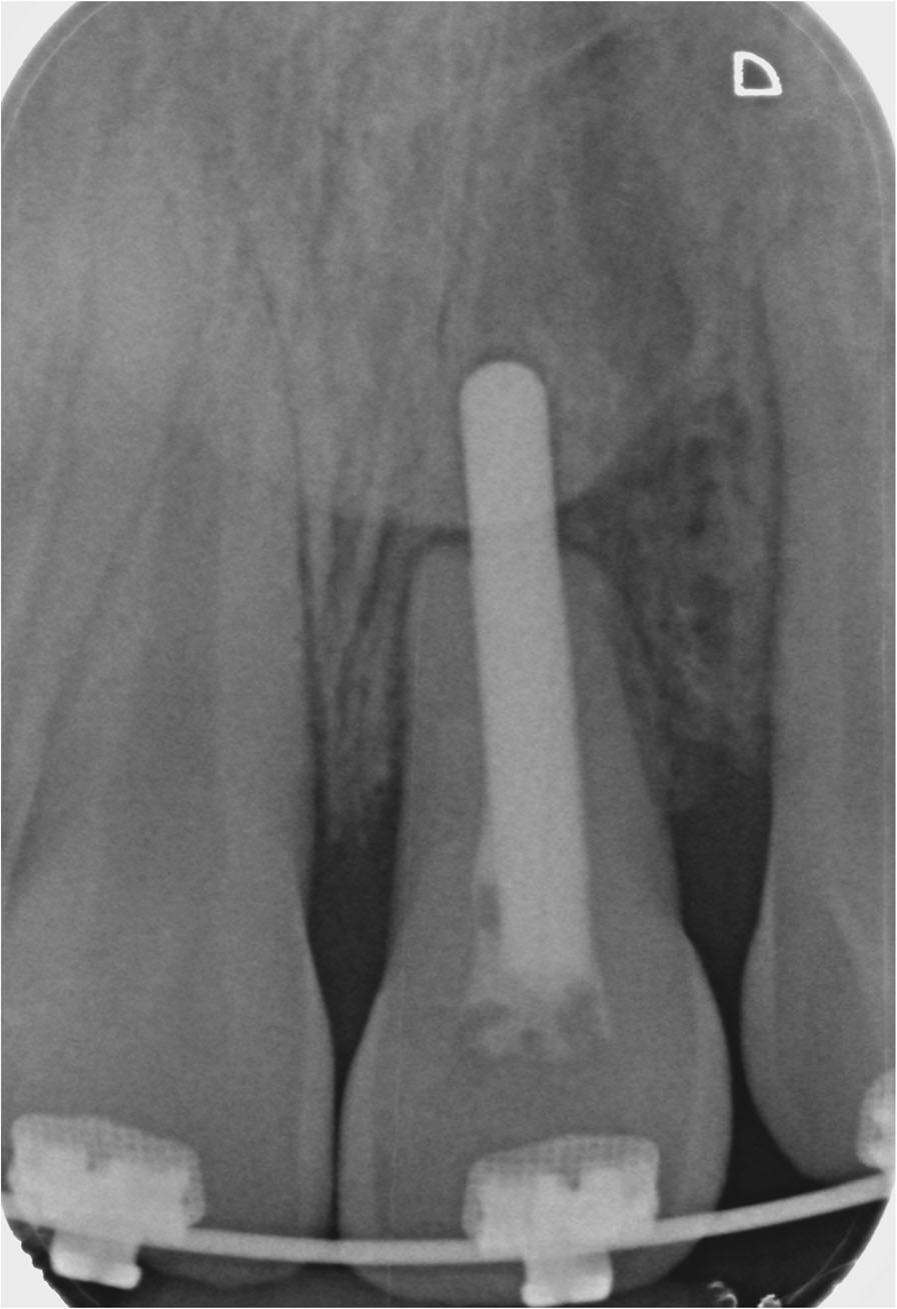
Radiographic control after 24 months

The transplantation of the incisor was performed using the standard treatment protocol according to Kirschner and the updated protocol by Pohl [13, 17]. The operation procedure included a careful preparation of the tooth so that the periodontal ligament could be preserved vital (Fig. 2). A dental splint fixed the tooth for 3 weeks. His postoperative course was uneventful and without complications. The orthodontic treatment to align the remaining teeth followed after 7 months. The transplanted central incisor was included in the fixed multibracket appliance and our patient did not show any atypical symptoms or reactions. In the follow-up 24 months later, the tooth was still functional without any clinical signs of pain or infection (Fig. 3). Radiography illustrated a reintegration of the root in the surrounding bone and the periodontal gap could be seen (Fig. 4 directly after transplantation, Fig. 5 after 24 months). No bone loss or reabsorption of the root in terms of replacement or infection reabsorption could be diagnosed. The soft tissue around his frontal teeth at the cleft site was stable; even his vestibule developed positively and was deeper than before.

The guidelines of the International Association of Dental Traumatology (IADT) for the success or failure of replantation describe the success criteria as clinically asymptomatic, normal mobility, no apical lesion in a radiograph, and no reabsorption or ankylosis of the root [18]. The presented case fits all the criteria; however, the mobility could not be tested due to orthodontic treatment.

## Discussion

One case report suggested secondary bone grafting with simultaneous auto-tooth transplantation as an option for alveolar cleft treatment [4]. In a larger case series, seven cases were presented that demonstrated the versatility of autotransplantation in a range of clinical situations. The authors noted that autotransplantation provides an excellent outcome in a growing child, with the advantage that it is a biologically compatible method of tooth replacement that promotes pulp and periodontal healing and enables orthodontic movement if necessary [5]. An interesting example of the transplantation of a canine to an incisor region with endodontic therapy and composite reshaping showed a successful follow-up over 2 years [5]. All these descriptions of treatment plans have one goal: to support the bone of the maxillary arch for the continuity of dental alignment. The maintenance of permanent teeth in the dental arch restores occlusal function, which contributes to the improvement of alveolar development in a growing patient [11]. The presented case has the same aim: to form a dental arch and preserve hard and soft tissues. We can recommend autotransplantation of incisors in patients with a cleft lip and alveolus to bridge the time until bone grafting or dental implantation becomes a viable option.

## Conclusions

Although these cases are rare, some publications report on the replantation of teeth in patients with a cleft lip and alveolus. Every case with a combination of cleft, tooth dislocation, or agenesis and compromised bone in the alveolar ridge presents a unique situation. Therefore, these cases require our special attention and an individual treatment plan. More publications offering a whole variety of treatment options are necessary in order for us to benefit from these unusual experiences.
